# Caerin 1.1 and 1.9 Peptides from Australian Tree Frog Inhibit Antibiotic-Resistant Bacteria Growth in a Murine Skin Infection Model

**DOI:** 10.1128/spectrum.00051-21

**Published:** 2021-07-14

**Authors:** Shu Chen, Pingping Zhang, Liyin Xiao, Ying Liu, Kuihai Wu, Guoying Ni, Hejie Li, Tianfang Wang, Xiaolian Wu, Guoqiang Chen, Xiaosong Liu

**Affiliations:** a Cancer Research Institute, Foshan First People’s Hospital, Foshan, Guangdong, China; b Clinical Microbiological Laboratory, Foshan First People’s Hospital, Foshan, Guangdong, China; c Genecology Research Centre, University of the Sunshine Coast, Maroochydore, QLD, Australia; d Department of Rheumatology, Foshan Frist People’s Hospital, Foshan, Guangdong, China; Lerner Research Institute

**Keywords:** MRSA, animal model, antibacterial, caerin peptides, natural antimicrobial agent, skin infection, soft tissue infection

## Abstract

The host defense peptide caerin 1.9 was originally isolated from skin secretions of an Australian tree frog and inhibits the growth of a wide range of bacteria *in vitro*. In this study, we demonstrated that caerin 1.9 shows high bioactivity against several bacteria strains, such as Staphylococcus aureus, Acinetobacter baumannii, methicillin-resistant Staphylococcus aureus (MRSA), and Streptococcus haemolyticus *in vitro*. Importantly, unlike the antibiotic Tazocin, caerin 1.9 does not induce bacterial resistance after 30 rounds of *in vitro* culture. Moreover, caerin 1.1, another peptide of the caerin family, has an additive antibacterial effect when used together with caerin 1.9. Furthermore, caerin 1.1 and 1.9 prepared in the form of a temperature-sensitive gel inhibit MRSA growth in a skin bacterial infection model of two murine strains. These results indicate that caerin 1.1 and 1.9 peptides could be considered an alternative for conventional antibiotics.

**IMPORTANCE** Antibiotic-resistant bacteria cause severe problems in the clinic. We show in our paper that two short peptides isolated from an Australian frog and prepared in the form of a gel are able to inhibit the growth of antibiotic-resistant bacteria in mice, and, unlike antibiotics, these peptides do not lead to the development of peptide-resistant bacteria strains.

## INTRODUCTION

Skin and soft tissue infections (SSTIs) are caused by pathogenic invasion of the skin and underlying soft tissues and have variable presentations, etiologies, and severities (1). The estimated incidence rate of SSTIs is 24.6 per 1,000 person-years (2), and approximately 70% to 75% of all cases are managed in the outpatient setting (3). Staphylococcus aureus is the leading cause of both uncomplicated and complicated infections. Moreover, multidrug-resistant bacteria, mainly methicillin-resistant S. aureus (MRSA; both community-acquired and health care-associated), are associated with significantly increased morbidity, mortality, length of hospital stay, and costs (4). SSTIs are generally managed in the community, including by self-application of antibiotics. Oral or systemic use of antibiotics is recommended at outpatient facilities or in hospitals, depending on the severity of the symptoms. Currently, topical application of antibiotics, such as mupirocin, is recommended for mild impetigo and folliculitis (5).

Due to the misuse and overuse of antibiotics, the emergence of antibiotic-resistant bacteria is becoming a serious health challenge (6). The rate of antimicrobial-resistant infections grows dramatically. Therefore, development of a novel and effective antibacterial agent is essential.

Naturally derived host defense peptides are one of the first successful forms of defense that eukaryotes have against bacteria, protozoa, fungi, and viruses. More than 200 host defense peptides have been isolated and identified from skin secretions of Australian frogs and toads. Many of these peptides, including caerin peptides, have antiviral, antitumor, antimicrobial, and/or neuropeptide-type activities (7–10). Caerin 1.1 and caerin 1.9 were originally isolated from the skin secretions of an Australian tree frog from the genus *Litoria*. The molecular weight of caerin 1.1 (GLLSVLGSVAKHVLPHVVPVIAEHL-NH_2_) and caerin 1.9 (GLFGVLGSIAKHVLPHVVPVIAEKL-NH_2_) were 2,584.12 and 2,593.17 g/mol, respectively (11). Caerin 1.1 has an anticancer effect against a number of human cancer cell lines, including leukemia, lung, colon, central nervous system (CNS), melanoma, ovarian, renal, prostate, and breast cancers (8, 12). Substitution of either or both of the Pro residues with Gly leads to a peptide with overall reduced activity (13). Both caerin 1.1 and caerin 1.9 peptides have antimicrobial activity against a wide spectrum of Gram-positive and Gram-negative microbial strains *in vitro* (7, 14–16). The caerin 1.1 and 1.9 peptides are heat resistant and are also stable under low pH (5.5 to 7.4) conditions and at room temperature (11). The caerin family of peptides are thought to interact with bacterial cell membranes by a carpet-like mechanism, whereby the peptides aggregate and orient themselves parallel to the membrane in a sheet-like arrangement followed by disruption of the bacterial cell membranes (13). Caerin 1.1 and 1.9 inhibit HIV-infected T cells within minutes after exposure at concentrations nontoxic to T cells and inhibit the transfer of HIV from dendritic cells (DCs) to T cells with limited toxicity (10, 17). Recently, it has been shown that caerin 1.1 and caerin 1.9 peptides have additive effects against human papillomavirus (HPV)-transformed tumor cells and increase the efficacy of a therapeutic vaccine against HPV-related diseases (9, 11, 18, 19).

In this study, we investigated whether the caerin 1.9 peptide is able to inhibit antibiotic-resistant bacteria growth and whether caerin 1.1 and caerin 1.9 have additive effects against bacteria growth *in vitro*. Their antibacterial ability was examined in a skin bacterial infection model *in vivo.*

## RESULTS

### MIC of caerin 1.9 against standard bacterial strains.

We first assessed the MICs of caerin 1.9 against S. aureus, P. aeruginosa, MRSA, A. baumannii, and S. haemolyticus. Bacteria in the logarithmic phase were cultured in the presence of different concentrations of caerin 1.9. The final concentrations of caerin 1.9 were 120, 60, 30, 15, 7.5, 3.75, and 1.875 μg/ml, respectively. The MIC was defined as the concentration of caerin 1.9 at which bacterial growth is completely inhibited. The MICs of caerin 1.9 against S. aureus, MRSA, A. baumannii, and S. haemolyticus were 7.5 μg/ml, while the MIC of caerin 1.9 against P. aeruginosa was 60 μg/ml (Table 1; see also Table S1 in the supplemental material).

**TABLE 1:** MIC (μg/ml) of caerin 1.9 against standard and clinically isolated bacterial strains

Standard strains					Clinically isolated strains		
*S. A* ^*a*^	*P. A*	MRSA	*A. B*	*S. H*	MRSA1	MRSA2	MRSA3
7.5	60	7.5	15	7.5	7.5	7.5	7.5

a *A. B*, A. baumannii; MRSA, methicillin-resistant S. aureus; *P. A*, P. aeruginosa; *S. A*, S. aureus; *S. H*, S. haemolyticus.

We next investigated the inhibitory effect of caerin 1.9 on clinically isolated MRSA. Patient information and antibiotic resistance of these bacteria strains are shown in Table S2. The MICs of caerin 1.9 against clinically isolated MRSA samples remained at 7.5 μg/ml (Table 1).

**FIG 1:**
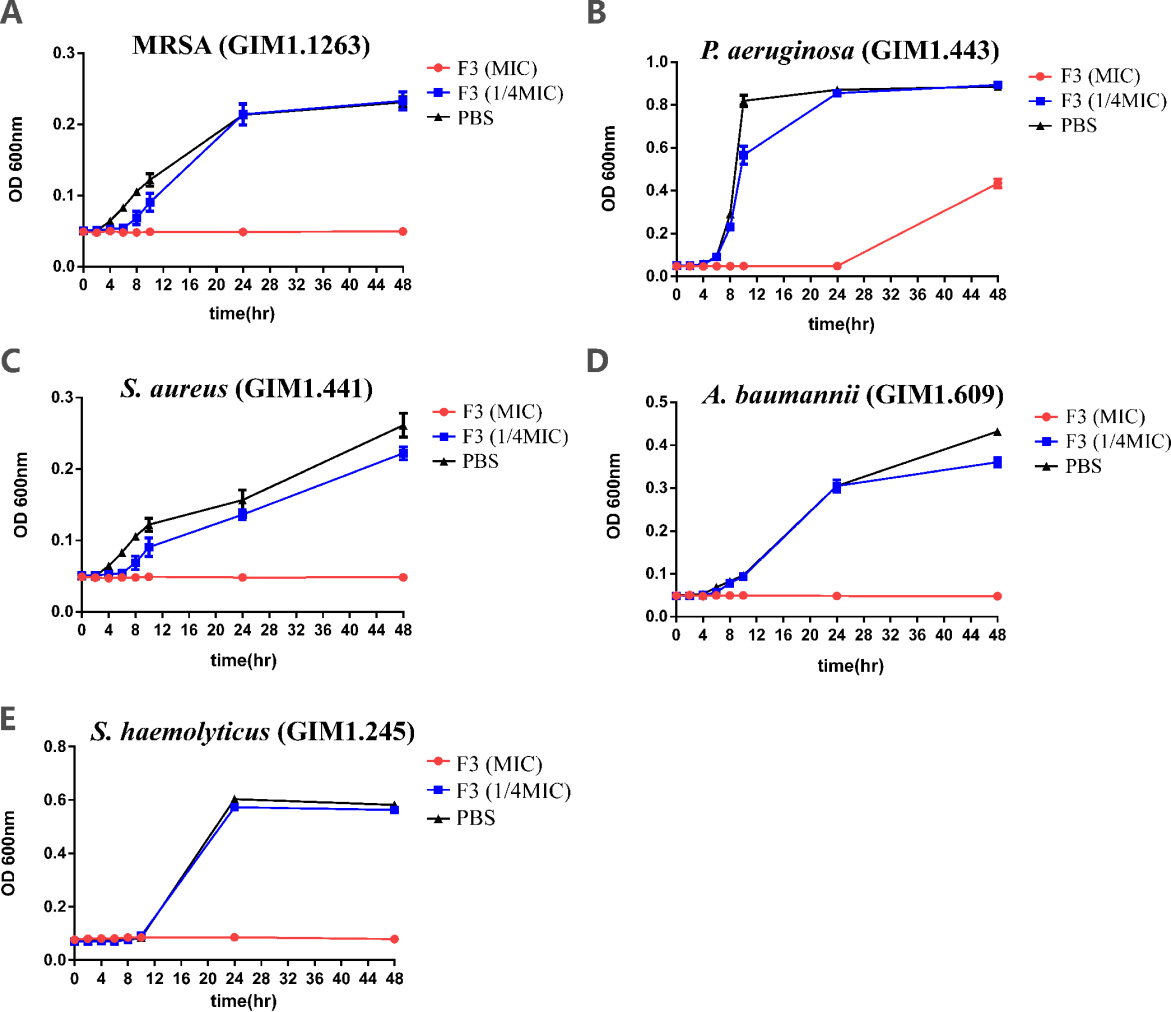
Change of optical density for MRSA (A), P. aeruginosa (B), S. aureus (C), A. baumannii (D), and S. haemolyticus (E). Different bacteria at an OD of 0.08 to 0.1 were cultured in medium with PBS or with caerin 1.9 at the MIC or at one-fourth the MIC. OD values were measured at 0, 2, 4, 6, 8, 10, 24, and 48 h by a UV spectrometer at a wavelength of 600 nm.

To better understand the dynamic inhibitory actions of caerin 1.9 against the growth of S. aureus, P. aeruginosa, MRSA, A. baumannii, and S. haemolyticus, these bacteria were then exposed to caerin 1.9 at the MIC or one-fourth of the MIC. When caerin 1.9 was cocultured with the bacteria at the MIC value, bacterial growth was completely inhibited in all tested bacteria for 24 h, except for P. aeruginosa. When the bacteria were cocultured with one-fourth of the MIC, the bacteria began to grow after 6 h, while the bacteria in the phosphate-buffered saline (PBS) group started to grow after 4 h. Meanwhile, the growth of S. haemolyticus was slower than other bacteria, which started increasing after 10 h of culture for both the PBS and one-fourth MIC groups. After 48 h of cultivation with the MIC, caerin 1.9 still inhibited S. aureus, MRSA, A. baumannii, and S. haemolyticus, but the inhibition of P. aeruginosa was poor (Fig. 1).

### Caerin 1.9 does not induce resistance strains.

Next, we investigated whether caerin 1.9 induces bioresistant strains after 30 passages. The logarithmic P. aeruginosa and MRSA were cultured in 96-well plates containing caerin 1.9 or piperacillin sodium (Tazocin sodium) at one-fourth the MIC. The medium was replaced every 3 days, and the whole culture process was continued for 3 months. After 3 months of culture with 30 passages, the MIC of caerin 1.9 against MRSA and P. aeruginosa did not change, suggesting no drug-resistant strain was induced. However, the MIC of the antibiotic Tazocin increased by 16-fold in MRSA and 8-fold in P. aeruginosa (Table 2).

**TABLE 2:** Comparison of caerin 1.9 and Tazocin on induced resistance of MRSA and Pseudomonas aeruginosa

Strains	Caerin 1.9 MIC^*a*^ (before)	Caerin 1.9 MIC (after)	Tazocin MIC (before)	Tazocin MIC (after)
MRSA (GIM1.1263)	7.5	7.5	7.5	120
P. aeruginosa (GIM1.443)	30	30	1.875	15

a MIC measured in μg/ml.

### Inhibition of S. aureus, MRSA, and S. haemolyticus by caerin 1.1/caerin 1.9 using the disk diffusion method.

As S. aureus, MRSA, and S. haemolyticus are the most commonly isolated bacterial strains during skin infection, the antibacterial ability of caerin 1.1 and 1.9 against bacterial strains that were identified mostly from skin infection were tested by using the disk diffusion method. Standard S. aureus, MRSA, and S. haemolyticus strains were used. Polymyxin B disks were used as a control, as it is an antibiotic used against skin infection that is capable of inducing a visible inhibition zone in all media at a dose of 120 μg or above (Table 3).

**TABLE 3:** Comparison of inhibition zone diameters among polymyxin B, caerin 1.1, and caerin 1.9 at a dose of 120 μg

	Polymyxin B^*a*^	Caerin 1.1	Caerin 1.9
MRSA	11.11 ± 0.135	14.62 ± 0.4208^*b*^	17.85 ± 0.5138^*c*^^,^^*d*^
S. aureus	11.59 ± 0.2539	15.29 ± 0.699^*e*^	17.79 ± 0.2982^*f*^^,^^*g*^
S. haemolyticus	11.93 ± 0.3269	12.22 ± 1.186	13.08 ± 1.025

a Paired *t* test was used to compare the caerin peptides with polymyxin B.

b,e Mean diameter of caerin 1.1 was significantly different from polymyxin B; *P* < 0.001.

c,f Mean diameter of caerin 1.9 was significantly different from polymyxin B; *P* < 0.0001.

d,g Significant difference between caerin 1.1 and caerin 1.9; *P* < 0.01.

The inhibition diameters of polymyxin B were consistent at 11 to 12 mm among three types of bacteria. Caerin 1.1 or caerin 1.9 developed larger inhibition zones, up to around 15 mm or 18 mm, against MRSA as well as S. aureus. For the inhibition zones developed in the S. haemolyticus medium, no significant difference between caerin 1.1 and polymyxin B or between caerin 1.9 and polymyxin B were found. The results suggested that both caerin 1.1 and caerin 1.9 have a better ability to inhibit the growth of S. aureus and MRSA than the commonly used antibiotic polymyxin B, but this was not significant against S. haemolyticus. Generally, caerin 1.9 exhibited stronger antimicrobial activity than caerin 1.1.

However, due to the positively charged property of caerin peptides, which renders an interaction between cationic polar headgroups and hydroxyl groups displayed on the disk paper, these peptides might exhibit reduced abilities to diffuse into the agar, making the antibacterial effect is unremarkable as demonstrated on agar plates (20, 21). Thus, the disk diffusion method may not reflect their actual ability in bacterial inhibition. Also, the disk diffusion method only provided a qualitative test for these peptides, which was not able to demonstrate the relationship between peptide concentrations and zone diameters.

### Caerin 1.1 and caerin 1.9 have additive effects against MRSA.

The combined bacterial growth inhibition effect of caerin 1.1 and caerin 1.9 on MRSA and A. baumannii was tested using the microdilution checkboard method (22). This method was based on the M38-A2 CLSI protocol for evaluating the bactericidal activity of the combination of caerin 1.1 and caerin 1.9 in different concentrations at 24 h. The *in vitro* interactions were calculated and interpreted as fractional inhibitory concentration index (FICI). The FICI against both bacteria was 0.5 < FICI ≤ 1, suggesting that they have an additive antibacterial effect on MRSA and A. baumannii (Table 4).

**TABLE 4:** Caerin 1.1 and caerin 1.9 combination against MRSA and Acinetobacter baumannii

	Single MIC (μg/ml)	Combined MIC (μg/ml)	FICI
MRSA			
Caerin 1.1	30	7.5	0.75
Caerin 1.9	7.5	3.75	
A. baumannii			
Caerin 1.1	7.5	0.9375	0.625
Caerin 1.9	3.75	1.875	

### Caerin 1.1 and 1.9 inhibit skin MRSA growth in mice.

**FIG 2:**
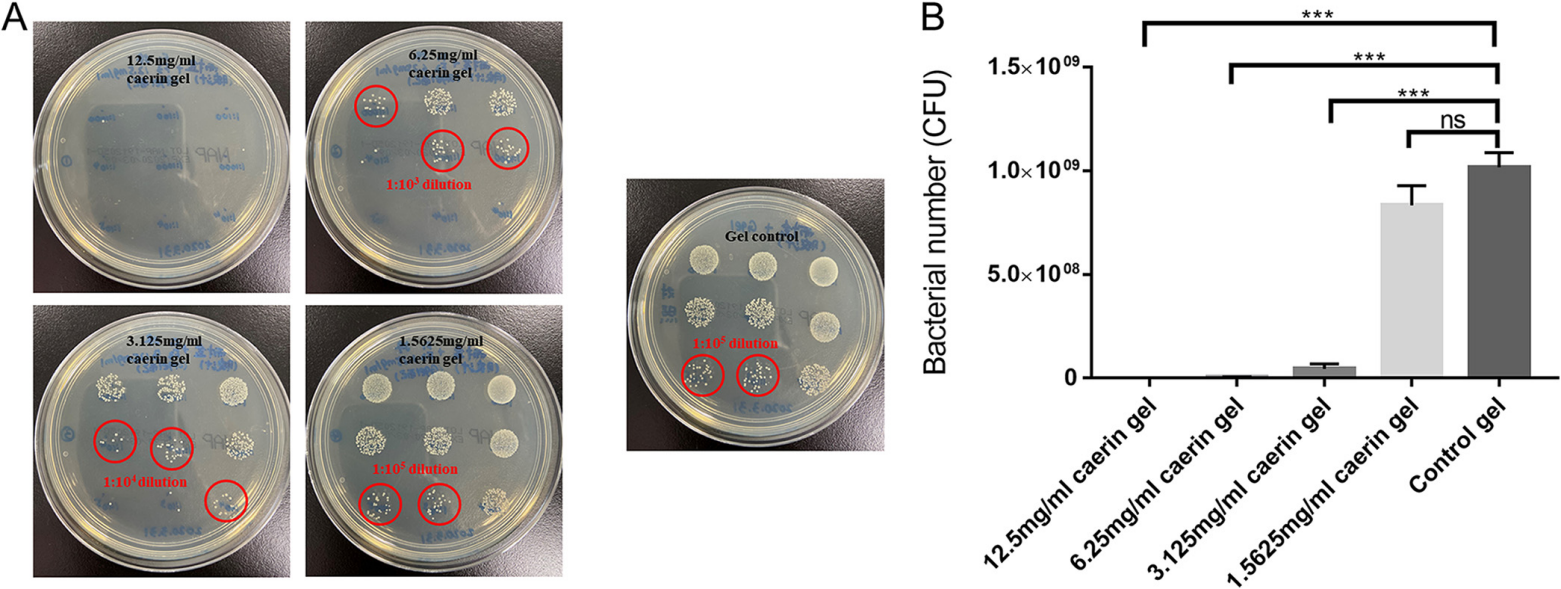
Caerin 1.1 and 1.9 gel inhibits the growth of MRSA from skin infection *in vitro*. (A) Pus resulting from MRSA-infected murine skin was mixed with different concentrations of caerin gel (12.5, 6.25, 3.125, and 1.5625 mg/ml) and control poloxamer gel, then 30 μl of the mixture was put onto a nutrition agar plate and incubated at 37°C for 24 h. (B) Each bar represents the number of countable bacterial colonies at certain concentrations, and the error bars represent the standard deviations. ***, *P* < 0.001; ns, not significant.

**FIG 3:**
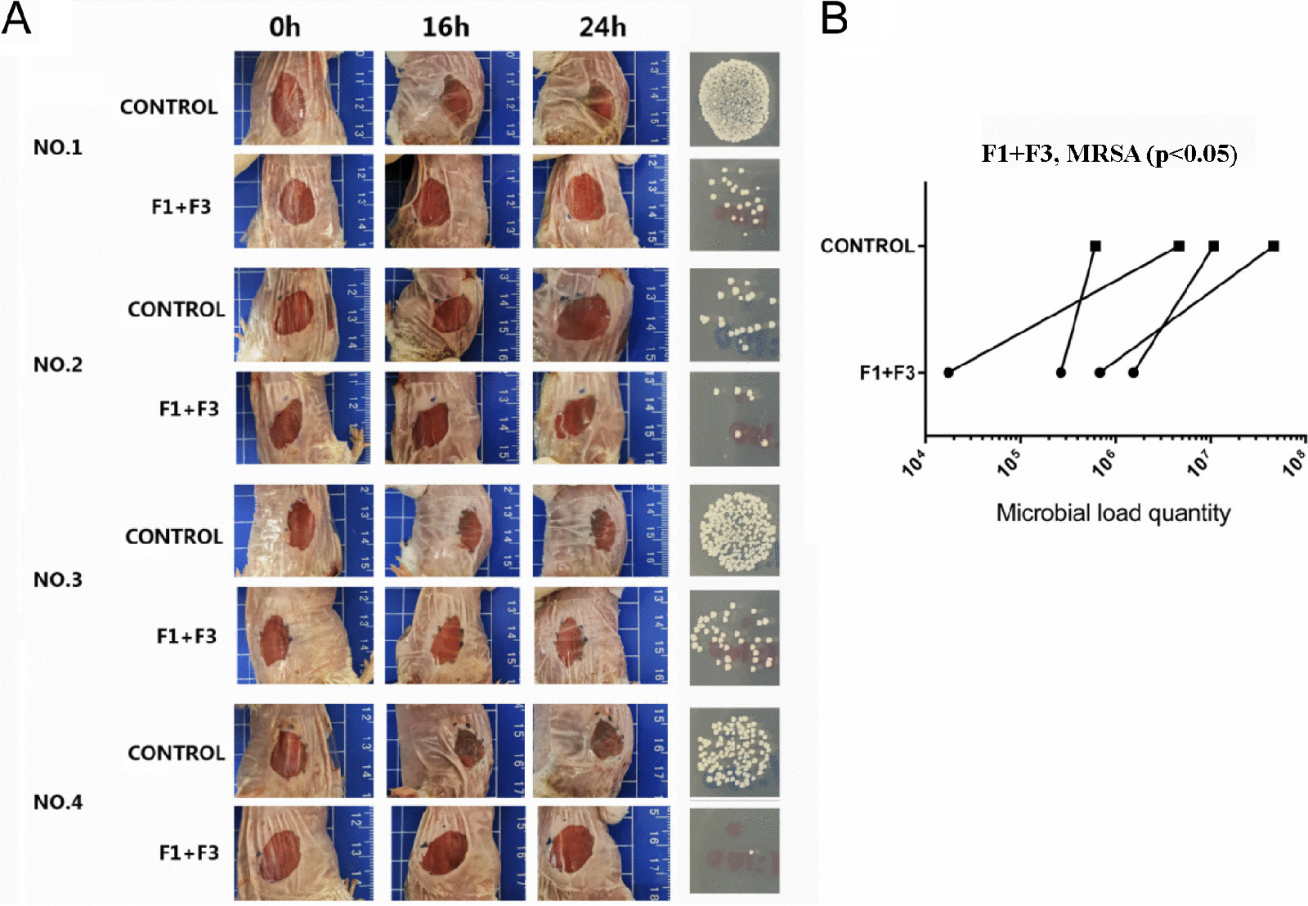
BALB/c mice with severe MRSA skin infection and the pair comparisons of bacteria counts between caerin gel and control gel. (A) Approximately 5 × 10^6^ CFU was inoculated on each wound with 12.5 mg/ml of caerin 1.1 (F1) and caerin 1.9 (F3) peptides in poloxamer gel or with gel only for 3 days. Four mice were included in this group. (B) The caerin gel inhibited the growth of MRSA at a significant level with a *P* value of <0.05.

Next, we investigated whether caerin 1.1 and caerin 1.9 inhibit bacteria growth in a skin MRSA infection model. We first tested whether the combinations of caerin 1.1 and caerin 1.9 (in a one-to-one molar ratio) inhibit the growth of MRSA isolated from a mouse skin infection site. Pus materials resulting from MRSA skin infection were mixed with different concentrations of caerin gel and inoculated onto a nutrition agar plate (LS0309, Guangzhou Dijing Microbiology Technology Co., Ltd.). After overnight incubation, the number of bacterial colonies indicated the *in vitro* antibacterial ability of caerin gel. At 1.5625 mg/ml, caerin gel started to inhibit the growth of MRSA, and the caerin gel almost eradicated the presence of MRSA at a concentration of 12.5 mg/ml (Fig. 2). Then, a severe skin damage model was established to ascertain that caerin peptide is able to achieve antibacterial activity *in vivo* (Fig. 3).

**FIG 4:**
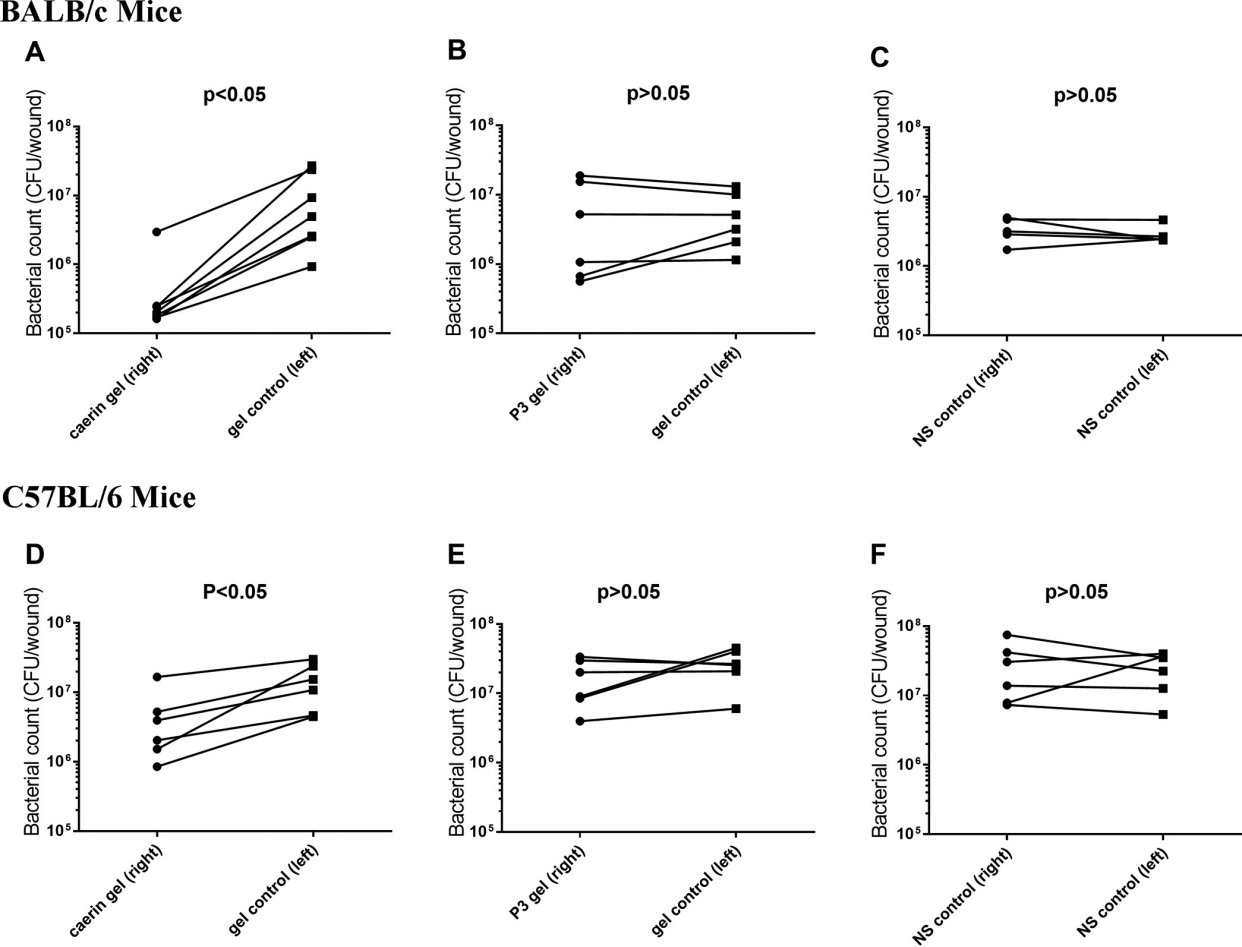
Caerin 1.1 and 1.9 gel inhibited the growth of MRSA (GIM1.1263), Tape-stripped BALB/c mice and C57BL/6 mice were infected with MRSA. Comparisons of bacteria counts between caerin gel (combined caerin 1.1 and caerin 1.9) and control gel, between P3 gel and control gel, and between two sides in the normal saline control group are shown. Approximately 5 × 10^4^ CFU was inoculated on each wound and treated with 12.5 mg/ml of caerin 1.1 and caerin 1.9 peptides in poloxamer gel or with gel only for 3 days. Each line represents the pair comparison of the number of bacteria (CFU) between the right- and left-stripped areas per mouse. In BALB/c mice, (A) the pair comparison of the CFU between caerin gel and control gel, (B) the pair comparison of the CFU between P3 gel and control gel, and (C) the pair comparison of the CFU between two sides of normal saline (NS) are depicted. Additionally, in C57BL/6 mice, (D) the pair comparison of the CFU between caerin gel and control gel, (E) the pair comparison of the CFU between P3 gel and control gel, and (F) the pair comparison of the CFU between two sides of normal saline are shown. The number of mice in each group was as follows: A, *n* = 7; B, *n* = 6; C, *n* = 5; D, *n* = 6; E, *n* = 6; F, *n* = 6. The results shown are representative of two independent experiments. Wilcoxon matched pairs test in the GraphPad Prism 7 software was used to analyze the data. The significance was determined by a *P* value at the level of 0.05.

The tape-stripped infection model was developed for investigating the therapeutic effect of caerin gel on MRSA-infected murine skin. The epidermal layer was damaged, allowing bacteria to colonize and grow on the skin surface as described in Materials and Methods (23). Four hours after infection, 20 μl of caerin gel or a control peptide P3 gel was applied to one infection area 2 times/day for 3 days, leaving another side with gel only. A group in which both damaged sides were treated with saline water was designed as a control. As a result, similar phenomena were observed in both BALB/c mice (Fig. 4A to C), C57BL/6 mice (Fig. 4D to F), and the severe MRSA skin infection model (Fig. 3). The saline water provided no therapeutic effect for MRSA infection (Fig. 4C and F). The bacterial counts of the caerin gel-treated areas were significantly reduced compared to the untreated areas (*P* < 0.05) (Fig. 4A and D). In the P3 gel group, the P3-treated and untreated areas had similar numbers of bacteria (Fig. 4B and E). These results suggested that the caerin peptide gel was effective in inhibiting bacterial growth for the superficial skin infection caused by MRSA.

## DISCUSSION

The higher incidence of skin and soft tissue infections (SSTIs) and the emergence of antibiotic-resistant bacteria require the development of novel alternatives against skin bacterial infections (6). Naturally derived antimicrobial peptides, such as caerin peptides, are potential candidates to overcome this challenge since they possess effective antibacterial activity against a broad spectrum of bacterial infections (7). Our previous studies report that caerin 1.9 and its combination with caerin 1.1 have potential in cancer therapy (18, 24). This study provides additional assessment for the bactericidal properties of caerin 1.9 combined with caerin 1.1 and its potential therapeutic effect on SSTIs. Our results suggested that the caerin 1.9 peptide exhibits effective antibacterial activity without inducing resistance after 30 passages, and caerin peptides in a temperature-sensitive gel formulation showed inhibitory effects against bacteria growth in the skin of MRSA-infected mice.

The antimicrobial activity of caerin 1.9 was tested against a range of Gram-positive and Gram-negative bacterial strains, including S. aureus, P. aeruginosa, MRSA, A. baumannii, and S. haemolyticus. The MIC values against various bacteria ranged from 3.75 to 15 μg/ml (except for P. aeruginosa), and caerin 1.9 was able to inhibit bacterial development at a lower concentration (Table 1; Fig. 1). Moreover, the disk diffusion method was used to compare the susceptibility of different bacterial strains to caerin peptides versus to polymyxin B. Currently, polymyxin B reemerged for the treatment of skin bacterial infections because of the high incidence of multidrug-resistant bacteria and lack of new antibacterial agents (25). However, it has limitations in treating certain Gram-positive strains tested in the current study, which required 120 μg or above to induce a visible bacterial growth inhibition zone on agar plates. Our results showed that both caerin 1.1 and caerin 1.9 have an edge over polymyxin B in inhibiting standard S. aureus, MRSA, A. baumannii, and S. haemolyticus (Table 3).

Caerin 1.1, one representative in the family of caerin peptides, has been demonstrated to have antimicrobial activity in multiple studies (13, 15, 26). We found that the combination of caerin 1.1 and caerin 1.9 exhibited an additive effect in inhibiting MRSA and Acinetobacter baumannii bacterial growth. Moreover, caerin 1.1 and 1.9 in a gel matrix formulation were able to inhibit MRSA growth *in vitro* starting from the concentration of 1.5625 mg/ml and displayed dose-dependent activity up to 12.5 mg/ml, where bacterial growth was completely inhibited (Fig. 2).

The superficial skin infection model was used to investigate the antibacterial activity of caerin gel *in vivo*. This model simulates SSTI in humans without disrupting the deeper layers of skin (23). It was convenient for evaluating externally applied antimicrobial agents as dermal antibiotic treatment. Mice presented with reduced genetic variations that might influence the results, therefore maximizing the reproducibility of the experiments (23, 27). The absorption of caerin gel depends on transdermal delivery. Transdermal delivery of therapeutic peptides permits the direct application to local infections and increases bioavailability by avoiding hepatic first-pass metabolism and gastrointestinal (GI) absorption (28). It reduces the dosing frequency, hence, lowering the cost and reducing adverse effects, providing patients with convenient and economical pain-free treatment.

Throughout the 3-day application of caerin gel on the damaged areas, caerin gel significantly inhibited the growth of MRSA in both BALB/c and C57BL/6 mice (Fig. 4) and in a more severe skin damage model (Fig. 3). Poloxamers, which compose the gel matrix, are commonly used surfactants in pharmaceuticals, which played a role in stabilizing or dispersing therapeutic agents to enhance dermal absorption. Thus, the poloxamer gel played a role in inhibiting *in vivo* MRSA growth by improving transdermal delivery in this study. The stability of the caerin gel was assessed by Ma et al. in our previous study (18). Although interindividual variations of sensitivity to the caerin therapy existed in the mice, the trend of reduced bacterial growth was observed. However, within the 3-day duration, it was not able to completely eradicate MRSA. Generally, the recovery of skin infection required a longer period; for example, the duration of treatment for impetigo lasted 7 to 14 days depending on severity (29). We are currently investigating the antibacterial activity of the caerin gel at lower concentrations and whether the caerin gel can completely clear the skin infection if applied to the skin infection site for a longer period.

Although the mouse model resembled human impetigo, some problems remained that impeded the direct application of the results to clinical practice. First of all, these mice were infected with a single bacterial strain, MRSA; but generally, patients in community or hospital settings might be infected with multiple bacterial strains at the same time (1). Additionally, the rate of elimination of microorganisms was faster in animals than in humans due to human complex physiological conditions (27). Therefore, our current results should be translated to realistic clinical trial in future studies.

The majority of caerin peptides display effective bactericidal activity through complementary mechanisms of action; therefore, they are capable of evading the development of resistance (30). Caerin peptides, including caerin 1.9, have similar primary structures based on that of caerin 1.1. Steinborner’s team highlighted that peptides in the caerin 1 family inhibit bacterial cell function with a similar mechanism but vary in selectivity toward different bacterial strains (16). The key action for bacterial cell disruption is dependent on membrane interaction, causing pore formation and membrane permeabilization (8, 31). The primary structure (more than 20 amino acids with two proline residues) of a caerin 1 peptide allows helical structure formation (15, 32). The α-helices aid the peptide in inserting to the lipid membrane for transmembrane pore formation through either barrel-stave mode or toroidal pore mode (20, 30, 33). Membrane permeabilization leads to the leakage of cell contents, imbalance of homeostasis, membrane dysfunction, and ultimately rapid lysis of bacterial cells. Caerin peptides exhibit alternative mechanisms depending on the peptide sequence as well as the concentration of peptide. At high concentrations, caerin 1 peptides may interact with lipid membrane by a carpet mechanism, which is more frequent in peptides with less than 20 amino acid residues (7, 33). The affinity and selectivity of peptide-membrane interactions are dependent on the compositions of the lipid membrane and concentration of peptides (31, 34). Therefore, the combined use of caerin 1.1 and caerin 1.9 may increase treatment efficacy and broaden the range of target bacteria.

As a positively charged peptide, caerin peptides can interact with bacterial membranes rather than normal mammalian cell membranes with special selectivity (30). Due to the different compositions and orientations of the lipid membrane, bacterial cells induce a stronger negative membrane potential than eukaryotic cells for peptide selectivity through electrostatic interaction, allowing caerin peptides to bind the bacterial cell membrane preferentially (35, 36). Also, high cholesterol contained in mammalian cell membranes aids in maintaining membrane stability, which is poor in bacterial cell membranes (30).

As discussed above, skin infection sometimes results from multibacterial infection. The treatment of mixed infections poses serious challenges in the clinic as they often organize themselves as a biofilm that is notoriously recalcitrant to antimicrobial therapy; however, antimicrobial peptides have the advantage of multiple functions, such as inhibition of bacterial adhesion, a wide spectrum of bacterial killing, quorum sensing, and the ability of inhibiting the production of extracellular polymeric substance production by bacteria (37). Therefore, it is worth investigating the possibility of using caerin 1.1 and caerin 1.9 against biofilm-related multibacterial infection.

In this study, we investigated the potential dermal application of caerin 1.1 and caerin 1.9 as a therapy for SSTIs. Nonetheless, some results must be interpreted with caution, and several limitations should be noted. Due to the restrictions of the MIC experiment, the tested MIC values might vary in every performance. Furthermore, neither the disk diffusion method nor the *in vivo* experiment demonstrates the dose-effect relationship of the antibacterial activity of caerin peptides against MRSA. We are currently using the tape-stripped infection model to determine the minimal dose of caerin peptide gel that inhibits MRSA growth *in vivo* and compare the bactericidal efficacy with other antibiotics, such as mupirocin and fusidic acid. Also, the potential toxicity and mechanism of action of the caerin peptides remain unknown in this study. However, this caerin gel, following further optimization, may provide an alternative method for the management of bacterial infection, especially for multidrug-resistant strains.

In conclusion, caerin 1.9 in combination with caerin 1.1 has the potential to become a novel antimicrobial agent because of its broad-spectrum antimicrobial activity and the limited emergence of bacterial resistance. The peptides in a gel formulation presented the therapeutic effect of inhibiting bacterial growth in animal models that simulated human skin bacterial infections. This caerin gel, following further optimization, may provide an alternative method for dermal treatment of skin and soft tissue infections.

## MATERIALS AND METHODS

### Mice.

Six- to 8-week-old, specific pathogen-free (SPF) adult female C57BL/6 (H-2^b^) mice and BALB/c mice were ordered from the Animal Resource Centre of Guangdong Province and kept at the Animal Facility of the Foshan First People’s Hospital, Foshan Guangdong, China. Experiments were approved and then performed in compliance with the guidelines of the Guangdong Animal Experimentation Ethics Committee (ethics approval number C202104-1). All mice were kept under clean conditions on a 12-h light/12-h dark cycle at 22°C with 75% humidity. Mice were provided with sterilized standard mouse food and water. Mice were given 1% sodium pentobarbital by intraperitoneal injection when treatment was performed. At the end of each experiment, mice were sacrificed by CO_2_ inhalation, which was confirmed by ceasing breath and heartbeat.

### Caerin peptide and caerin gel preparation.

Caerin 1.1 (F1) and caerin 1.9 (F3) peptides were derived from the Australian tree frog Litoria splendida. Caerin 1.1 (GLLSVLGSVAKHVLPHVVPVIAEHL-NH_2_), caerin 1.9 (GLFGVLGSIAKHVLPHVVPVIAEKL-NH_2_), and a control peptide that does not have cytotoxic properties against a variety of cancerous cells (GTELPSPPSVWFEAEFK-OH) (9) were synthesized by Mimotopes Proprietary Limited in Wuxi, China. The purity of the peptides was >95% as determined by reverse-phase high-performance liquid chromatography (HPLC) at Mimotopes. The lipopolysaccharide concentration of caerin 1.1, caerin 1.9, and control peptide (P3) was less than 0.44 EU/ml as measured by a kinetic turbidimetric assay by Xiamen Bioendo Technology Co., Ltd.

Poloxamer 407 (molecular weight 12,600 Dalton, batch number WPAK592B) and poloxamer 188 (molecular weight 8,400 Dalton, batch number WPAK539B) were purchased from Badische Anilin- und SodaFabrik (BASF; Ludwigshafen, Germany).

The gel matrix was prepared by mixing 46 g of poloxamer 407 and 10 g of poloxamer 188 with 200 ml of distilled water. The preparation was stirred until a white condensation gum matrix was formed and stored at 4°C until the poloxamers were completely dissolved. The caerin peptide gel was prepared by mixing caerin 1.1 and 1.9 with the gel matrix. After the peptides were completely dissolved, the solution was filtered through a 0.22-μm microporous membrane filter to prepare the caerin gel. P3 gel was prepared similarly. The caerin gel and P3 gels were stored at 4°C until use.

### Bacteria.

Standard strains of Staphylococcus aureus (S. aureus, GIM1.441), copper-green Pseudomonas (P. aeruginosa; GIM1.443), methicillin-resistant Staphylococcus aureus (MRSA; GIM1.1263), baumann (Acinetobacter baumannii; GIM1.609), and Streptococcus haemolyticus (S. haemolyticus; GIM1.245), were purchased from the Guangdong Microbial Species Conservation Center, Guangdong, China.

Clinical strains of methicillin-resistant Staphylococcus aureus were isolated and identified from patient specimens at the Foshan First People’s Hospital (Table S3 in the supplemental material) by the Department of Pathology of the hospital.

### Bacterial strain resuscitation and preservation.

The freeze-dried purchased bacteria were added into 5 ml of Mueller-Hinton (MH) broth medium (3.65 g of tryptone purchased from Guangdong Ring Kai Microbiology Technology Co., Ltd., dissolved in 100 ml of distilled water, 121°C, autoclaved for 20 min) and cultured at 37°C for 24 h. The bacterial suspensions were then inoculated onto a nutrition agar plate (LS0309, Guangzhou Dijing Microbiology Technology Co., Ltd.) and incubated at 37°C overnight. The bacterial colonies were picked up using sterile filter paper and stored at −80°C.

### Establishing the standard curve of bacterial concentration and light optical density.

MRSA (GIM1.1263) in the logarithmic growth stage was centrifuged at 8,000 rpm for 2 min, washed with phosphate-buffered saline, then resuspended in MH medium and inoculated onto a nutrition agar plate (LS0309, Guangzhou Dijing Microbiology Technology Co., Ltd.) to determine the bacteria concentration by counting the colonies. The MRSA suspension was serially diluted, and the absorbance was measured by an ultraviolet (UV) spectrophotometer (UV-7504, Xin Mao, Shanghai, China) at a wavelength of 600 nm. The correlation of bacterial concentration and the value of photodensity was established using a linear regression method (Fig. S1).

### MIC of caerin 1.9.

The MICs of caerin 1.9 were measured by using a microbroth dilution method developed by the CLSI (38). The bacterial suspension with absorbance ranging from 0.08 to 0.10 was prepared, and 100 μl of the bacterial suspension was added to a 96-well U-shaped culture plate, followed by adding 100 μl of different concentrations of caerin 1.9. The final concentrations of caerin 1.9 were 120, 60, 30, 15, 7.5, 3.75, and 1.875 μg/ml, respectively. Each concentration of caerin 1.9 was added in triplicate. The equivalent volume of PBS was added as a growth control. The bacteria were cultured at 37°C for 24 h. The MIC is defined as the concentration of peptide where bacterial growth is completely inhibited.

### Dynamic bacterial inhibition assay.

Bacterial suspensions (100 μl) at an optical density (OD) value of 0.08 to 0.1 were added to 96-well U-shaped plates, followed by adding the same amount of caerin 1.9 solution in triplicate. The final caerin 1.9 concentration was either the MIC value or one-fourth of the MIC value. PBS was added as a bacterial growth control group. The bacteria were cultured at 37°C for 48 h, and the concentrations were measured at multiple time points by an enzyme-linked immunosorbent assay (ELISA) plate reader (Multiskan Go, Thermo Scientific, Waltham, MA, USA) at an OD of 600 nm.

### Drug resistance induction.

P. aeruginosa and MRSA were prepared by dilution and then cultured in a 96-well U-shaped plate containing one-fourth of the MIC value of caerin 1.9, Tazocin, or PBS. Every 3 days, the cultured bacteria were transferred to new medium containing the same amount of caerin 1.9 or Tazocin. After 30 rounds of culture, the MIC values of caerin 1.9 or Tazocin against the two bacteria were measured as described above. The MIC values before and after the treatment were compared.

### Antimicrobial susceptibility test of caerin 1.1 and caerin 1.9.

The antimicrobial activity of caerin 1.1 and caerin 1.9 were evaluated using the disk diffusion method according to the guidelines of the CLSI (39). First, the nutrition agar plates were inoculated with standardized bacteria strains. Then, 6-mm filter paper disks, which were carpeted with 120 μg of caerin 1.1, caerin 1.9, or polymyxin B, were placed on the agar surface. The plates were cultured at 37°C for 24 h to allow for the formation of the inhibition growth zone. The inhibition zone diameter was measured using a vernier caliper.

### Caerin 1.1 and caerin 1.9 combination against MRSA and A. baumannii.

The combination of caerin 1.1 and caerin 1.9 against MRSA and A. baumannii was tested with a microdilution checkerboard assay described elsewhere (22). First, the serially diluted bacterial suspensions were cultured in a 96-well U-shaped culture plate in the presence of caerin 1.1 and 1.9. The final concentrations of caerin 1.1 and 1.9 in the plate were from 2 times the MIC to l/64 of the MIC. For wells along the *x* axis, caerin 1.9 was added, while caerin 1.1 was added along the *y* axis. Each well within this 8 × 8 checkerboard contained a combination of caerin 1.1 and 1.9 at different concentrations. The fractional inhibitory concentration index (*FICI*) for a well was calculated as

$$FICI=FIC1.1+FIC1.9=\frac{A}{MIC1.1}\;+\;\frac{B}{MIC1.9}$$

Here, *A* is the concentration of caerin 1.1 in a given well along the inhibition-no inhibition interface, *MIC*1.1 is the MIC of caerin 1.1 alone, *FIC*1.1 is the fractional inhibitory concentration of caerin 1.1, and *B*, *MIC*1.9, and *FIC*1.9 are defined in the same fashion for caerin 1.1. A *FICI* of ≤1.0 implies an additive effect or synergy.

### Skin infection model.

Six- to 8-week-old female BALB/c mice with their fur on the back removed were anesthetized and the skin was cut into an oval shape of around 1 cm × 2 cm. A bacterial infection was initiated by placing MRSA suspension (containing 5 × 10^6^ CFU) onto each damaged area, followed by application of 20 μl of caerin gel to the left side of the stripped area and 20 μl of poloxamer gel to the right side. The treatment was performed twice at 4 h and 16 h, respectively. At 24 h, pus and secretions on each damaged area were collected, diluted serially with normal saline, and then inoculated onto a nutrition agar plate for culture overnight. The colonies were counted and compared.

### Tape-stripping infection model.

A tape-stripping infection model was used to investigate the antibacterial ability of caerin 1.1 and 1.9 gel (23). Briefly, 6- to 8-week-old female BALB/c mice or C57BL/6 mice were anesthetized. After the removal of the fur on the back, the skin was stripped with an elastic bandage (Smith & Nephew Medical, Hull, UK). Two areas of 1 cm × 2 cm on both left and right sides of the torso were tape stripped. After stripping, 10 μl of MRSA suspension (GIM1.1263; 5 × 10^6^ CFU/ml) was applied to each stripped surface. Then, 4 h after the skin infection, 20 μl of caerin 1.1 and 1.9 gel containing 12.5 mg/ml of each peptide was applied to the left side of the stripped area, while the same amount of poloxamer gel was applied to the right side. In another group, 20 μl of P3 gel at 12.5 mg/ml was applied to the left, and poloxamer gel was applied to the right. Mice in the infection control group were given 20 μl of normal saline. Each treatment was performed twice daily for 3 consecutive days for a total of five times. On the fourth day, the stripped areas were removed, subsequently homogenized by a glass homogenizer, and diluted with normal saline, followed by inoculation on nutrition agar plates (LS0309, Guangzhou Dijing Microbiology Technology Co., Ltd.) for culture for 24 h before the colonies were counted.

### Statistical analysis.

Paired Student’s *t* test statistical analysis was performed to evaluate *in vivo* bacterial growth inhibition using GraphPad Prism 7 software. All experimental data were analyzed and graphs plotted in the same software. The significant means were determined at the probability level of 0.05.
