# Supplementary material for: Caerin 1.1 and 1.9 Peptides from Australian Tree Frog Inhibit Antibiotic-Resistant Bacteria Growth in a Murine Skin Infection Model
Source: Microbiol Spectr. 2021 Jul 14;9(1):10.1128/spectrum.00051-21. doi: 10.1128/spectrum.00051-21 (PMC8552723; doi:10.1128/spectrum.00051-21)

Table S1. List of minimum inhibitory concentration MIC (µg/ml) of caerin1.9 and caerin 1.1 against different bacteria

		**MIC of Caerin 1.9**	**MIC of Caerin 1.1**
Staphylococcus aureus (*S. Aureus*, GDM1.441)	2019-03-18		15
	2019-03-19		15
	2019-04-02	30	
	2019-07-18	7.5	15
	2019-07-18	7.5	
	2019-07-23	7.5	
	2019-07-24	7.5	
	2019-08-19	3.75	
	2020-01-27	7.5	15
		**7.5**[a]	15
Methicillin-resistant *Staphylococcus aureus* (MRSA, GDM1.1263)	2019-06-27		30
	2019-04-08	7.5	30
	2019-07-23	7.5	
	2019-07-24	7.5	
	2019-08-19	7.5	
	2019-08-26	3.75	
	2020-01-27	15	30
		**7.5**[b]	30
Pseudomonas aeruginosa (*P. aeruginosa*, GDM1.443)	2019-05-07	60	60
	2019-08-09	60	60
	2019-08-19	30	
		**60**[c]	60
Acinetobacter Baumannii (*A. Baumannii* GDM1.609)	2019-08-22	15	
	2019-08-22	15	
	2019-08-26	3.75	
		**15**[d]	
Streptococcus hemolyiicus (*S. hemolyiicus*, GDM1.245)	2020-01-14	15	30
	2020-01-26	7.5	15
	2020-01-27	7.5	15
		**7.5**[e]	15

[a, b, c, d, e] mode was selected to represent the results.

Table S2. Caerin peptide stability test. Minimum inhibitory concentration MIC (µg/ml) of caerin1.9 and caerin 1.1 against MRSA after interventions of temperature and pH

		**MIC of Caerin 1.9**	**MIC of Caerin 1.1**
Temperature **effect**[a]	-40°C (control)	7.5	30
	4°C	7.5	60
	25°C	7.5	30
	37°C	7.5	30
pH **effect**[b]	pH=3	7.5	30
	pH=4	15	60
	pH=5	7.5	30
	pH=6	7.5	60
	pH=7	7.5	30
	pH=8	7.5	30
	pH=9	3.75	30
	pH=10	7.5	30
	pH=11	7.5	60

[a] As temperature change, the antibacterial activity of caerin 1.1 and caerin 1.9 are stable with minimal fluctuations. Caerin peptides were cultured in PBS at various temperature (-40, 4, 25, 60°C) for 4 days before the MIC experiment was performed. PBS treatment served as a control. Each sample was performed in triplicate.

[b] pH change did not alter the antibacterial activity of caerin 1.1 and caerin 1.9. The MIC values only fluctuated in a narrow range. Caerin peptides were culture in PBS at various pH environment (from 3 to 11) for 2 hours before the MIC experiment was performed. PBS treatment served as a control. Each sample was performed in triplicate.

Table S3. Detailed Patient Information of Clinical isolated MRSA samples and Results of Clinical Antimicrobial Susceptibility Test

Clinically isolated MRSA strains		MRSA Sample 1	MRSA Sample 2	MRSA Sample 3
MIC (μg/ml)	Caerin 1.9	7.5	7.5	7.5
	Caerin 1.1	60	15	30
	Tazocin	>30	>30	>30
Clinical Antimicrobial Susceptibility Test for Different Drugs	Penicillin G	R	R	R
	Ceftaroline fosamil	/	/	S
	Rifampin	S	S	S
	Levofloxacin	/	/	S
	Clindamycin	R	R	R
	Erythromycin	R	R	S
	Vancomycin	S	S	S
	Tigecycline	/	S	S
	Oxacillin	R	R	R
	Gentamicin	R	S	S
	Moxifloxacin	R	S	R
	SMZ-TMP	/	/	S
	Daptomycin	/	/	S
	Linezolid	S	S	S
	Teicoplanin	/	/	S
	Novobiocin	S	/	S
MASA Identification	Screening of Cefoxitin	(+)	(+)	(+)
	β-lactamase	(+)	(+)	(+)
	Induction of Clindamycin	(-)	(-)	(-)
Diagnosis		Cerebral Infraction	Pelvic cyst	Type 2 diabetes
Sample Source		Sputum	Puncture fluid	Wound secretion

Figure S. The correlation of the concentration of bacterial suspension and optical density at the wavelength of 600nm. The equation and R-square were calculated by linear regression.

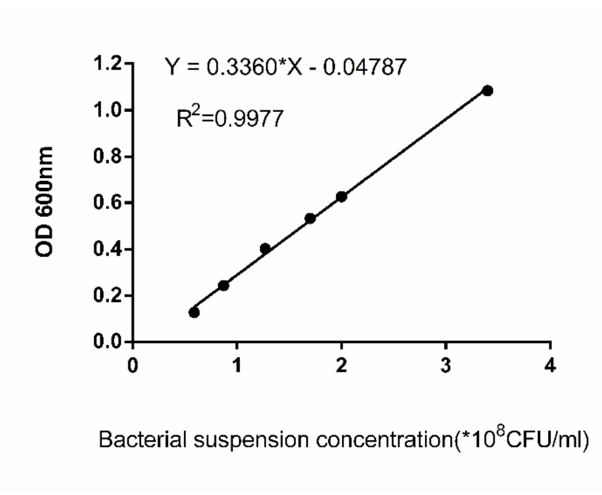

Supplement: SUPPLEMENTAL FILE 1 — Supplemental material. Download SPECTRUM00051-21_Supp_1_seq8.pdf, PDF file, 0.2 MB [file spectrum00051-21_supp_1_seq8.pdf]
